# Photoperiod and temperature as dominant environmental drivers triggering secondary growth resumption in Northern Hemisphere conifers

**DOI:** 10.1073/pnas.2007058117

**Published:** 2020-08-05

**Authors:** Jian-Guo Huang, Qianqian Ma, Sergio Rossi, Franco Biondi, Annie Deslauriers, Patrick Fonti, Eryuan Liang, Harri Mäkinen, Walter Oberhuber, Cyrille B. K. Rathgeber, Roberto Tognetti, Václav Treml, Bao Yang, Jiao-Lin Zhang, Serena Antonucci, Yves Bergeron, J. Julio Camarero, Filipe Campelo, Katarina Čufar, Henri E. Cuny, Martin De Luis, Alessio Giovannelli, Jožica Gričar, Andreas Gruber, Vladimír Gryc, Aylin Güney, Xiali Guo, Wei Huang, Tuula Jyske, Jakub Kašpar, Gregory King, Cornelia Krause, Audrey Lemay, Feng Liu, Fabio Lombardi, Edurne Martinez del Castillo, Hubert Morin, Cristina Nabais, Pekka Nöjd, Richard L. Peters, Peter Prislan, Antonio Saracino, Irene Swidrak, Hanuš Vavrčík, Joana Vieira, Biyun Yu, Shaokang Zhang, Qiao Zeng, Yaling Zhang, Emanuele Ziaco

**Affiliations:** ^a^Key Laboratory of Vegetation Restoration and Management of Degraded Ecosystems, South China Botanical Garden, Chinese Academy of Sciences, Guangzhou 510650, China;; ^b^Center of Plant Ecology, Core Botanical Gardens, Chinese Academy of Sciences, Guangzhou 510650, China;; ^c^Département des Sciences Fondamentales, Université du Québec à Chicoutimi, Chicoutimi, QC G7H 2B1, Canada;; ^d^DendroLab, Department of Natural Resources and Environmental Science, University of Nevada, Reno, NV 89557;; ^e^Dendrosciences, Swiss Federal Research Institute for Forest, Snow and Landscape, CH-8903 Birmensdorf, Switzerland;; ^f^Key Laboratory of Alpine Ecology and Biodiversity, Key Laboratory of Tibetan Environment Changes and Land Surface Processes, Institute of Tibetan Plateau Research, Chinese Academy of Sciences, Beijing 100101, China;; ^g^Department of Forests, Natural Resources Institute Finland, 02150 Espoo, Finland;; ^h^Department of Botany, Leopold-Franzens University of Innsbruck, 6020 Innsbruck, Austria;; ^i^AgroParisTech, Institut National de Recherche pour l’Agriculture, l’Alimentation et l’Environnement, Université de Lorraine, Silva, F-54000 Nancy, France;; ^j^Dipartimento di Agricoltura, Ambiente e Alimenti, Università degli Studi del Molise, 86100 Campobasso, Italy;; ^k^Department of Physical Geography and Geoecology, Charles University, CZ-12843 Prague, Czech Republic;; ^l^Cold and Arid Regions Environmental and Engineering Research Institute, Chinese Academy of Sciences, Lanzhou 730000, China;; ^m^Chinese Academy of Sciences Key Laboratory of Tropical Forest Ecology, Xishuangbanna Tropical Botanical Garden, Chinese Academy of Sciences, Mengla, Yunnan 666303, China;; ^n^Forest Research Institute, Université du Quebec en Abitibi-Témiscamingue, Rouyn-Noranda, QC J9X5E4, Canada;; ^o^Instituto Pirenaico de Ecología, Consejo Superior de Investigaciones Científicas, 50192 Zaragoza, Spain;; ^p^Centre for Functional Ecology, Department of Life Sciences, University of Coimbra, 3000-456 Coimbra, Portugal;; ^q^Biotechnical Faculty, University of Ljubljana, 1000 Ljubljana, Slovenia;; ^r^Department of Forest and Carbon Resources, Institut National de Information Géographique et Forestière (IGN), 54250 Champigneulles, France;; ^s^Department of Geography and Regional Planning, Environmental Science Institute, University of Zaragoza, 50009 Zaragoza, Spain;; ^t^Istituto di Ricerca sugli Ecosistemi Terrestri, Consiglio Nazionale delle Ricerche, 50019 Sesto Fiorentino, Italy;; ^u^Laboratory for Dendrochronology, Slovenian Forestry Institute, 1000 Ljubljana, Slovenia;; ^v^Department of Wood Science and Wood Technology, Mendel University in Brno, 61300 Brno, Czech Republic;; ^w^Institute of Botany, University of Hohenheim, 70593 Stuttgart, Germany;; ^x^Department of Biology, Southwest Anatolia Forest Research Institute, 07010 Antalya, Turkey;; ^y^State Key Laboratory for Conservation and Utilization of Subtropical Agro-bioresources, College of Life Sciences, South China Agricultural University, Guangzhou 510642, China;; ^z^Department of Sciences, University of Alberta, Camrose, AB T4V 2R3, Canada;; ^aa^Key Laboratory of Aquatic Botany and Watershed Ecology, Wuhan Botanical Garden, Chinese Academy of Sciences, Wuhan 430074, China;; ^bb^Dipartimento di Agraria, Università Mediterranea di Reggio Calabria, 89122 Reggio Calabria, Italy;; ^cc^Laboratory of Plant Ecology, Department of Plants and Crops, Faculty of Bioscience Engineering, Ghent University, B-9000 Ghent, Belgium;; ^dd^Department of Agricultural Sciences, University of Naples Federico II, I-80055 Portici-Napoli, Italy;; ^ee^Key Laboratory of Guangdong for Utilization of Remote Sensing and Geographical Information System, Guangdong Open Laboratory of Geospatial Information Technology and Application, Guangzhou Institute of Geography, Guangzhou 510070, China

**Keywords:** xylogenesis, wood formation, photoperiod, temperature, Northern Hemisphere conifer

## Abstract

Wood formation consumes around 15% of the anthropogenic CO_2_ emissions per year and plays a critical role in long-term sequestration of carbon on Earth. However, the exogenous factors driving wood formation onset and the underlying cellular mechanisms are still poorly understood and quantified, and this hampers an effective assessment of terrestrial forest productivity and carbon budget under global warming. Here, we used an extensive collection of unique datasets of weekly xylem tissue formation (wood formation) from 21 coniferous species across the Northern Hemisphere (latitudes 23 to 67°N) to present a quantitative demonstration that the onset of wood formation in Northern Hemisphere conifers is primarily driven by photoperiod and mean annual temperature (MAT), and only secondarily by spring forcing, winter chilling, and moisture availability. Photoperiod interacts with MAT and plays the dominant role in regulating the onset of secondary meristem growth, contrary to its as-yet-unquantified role in affecting the springtime phenology of primary meristems. The unique relationships between exogenous factors and wood formation could help to predict how forest ecosystems respond and adapt to climate warming and could provide a better understanding of the feedback occurring between vegetation and climate that is mediated by phenology. Our study quantifies the role of major environmental drivers for incorporation into state-of-the-art Earth system models (ESMs), thereby providing an improved assessment of long-term and high-resolution observations of biogeochemical cycles across terrestrial biomes.

## Significance

Forest trees can live for hundreds to thousands of years, and they play a critical role in mitigating global warming by fixing approximately 15% of anthropogenic CO_2_ emissions annually by wood formation. However, the environmental factors triggering wood formation onset in springtime and the cellular mechanisms underlying this onset remain poorly understood, since wood forms beneath the bark and is difficult to monitor. We report that the onset of wood formation in Northern Hemisphere conifers is driven primarily by photoperiod and mean annual temperature. Understanding the unique relationships between exogenous factors and wood formation could aid in predicting how forest ecosystems respond and adapt to climate warming, while improving the assessment of long-term and high-resolution observations of global biogeochemical cycles.

Forests cover 31% of the Earth’s land surface and play critical ecological and economic roles in regulating global carbon, water, and energy cycles (1). Over the past decades, trees have sequestered approximately one-third of the anthropogenic carbon dioxide emissions through cyclical and tightly coordinated growth of primary and secondary meristems, thereby serving as major long-term terrestrial biotic carbon sinks (2). However, recent climate warming has changed the seasonal timing of the primary (budburst, leafing, and flowering) and secondary (cambial activity and xylem and phloem formation) growth of trees (3, 4). These changes could have potentially dire but as-yet-unidentified consequences for forest productivity and ecosystem structure and functioning, as well as carbon and energy cycles.

A widespread advancement has been documented in the springtime phenology of tree primary growth in response to recent warming (3, 4). The result has been a greater synchronization of springtime phenological events along latitudinal (5) or altitudinal (6) gradients toward higher latitudes and altitudes. The mechanisms underlying this skewed synchronism are largely attributed to changes in winter chilling (i.e., the sum of low-temperature incidents required to cause rest break in the buds, henceforth “chilling”) and spring forcing requirements (i.e., the sum of temperatures above a specific threshold required to cause ontogenetic development toward bud burst, henceforth “forcing”) (7). These changes have resulted from an asymmetric warming across time (seasons and years) and over space (altitude and latitude) (5, 6). Temperature plays a further dominant role in triggering primary growth during both endodormancy and ecodormancy phases (8). However, photoperiod (or daylength, which was calculated for the site locations as the time interval between sunrise and sunset on the onset day of wood formation of each individual tree per year in our study) is also decisive in controlling dormancy induction and release, growth initiation, and reproductive events (9). For this reason, photoperiod and its interaction with temperature are widely recognized as regulators of primary and secondary tree growth phenology (9, 10), but their synergistic effects have rarely been quantified.

Overall, the mechanisms behind the recent warming-induced changes in the springtime phenology of primary growth (mostly focused on broadleaf species) are becoming increasingly well understood and elucidated (11). By contrast, secondary growth phenology (henceforth, specifically referred to as wood formation onset) remains less well understood, although advancements in xylem phenology are now enabling precise and frequent monitoring of cambium cell differentiation and wood formation (i.e., xylogenesis) (12). Over the past two decades, studies on secondary growth phenology (mostly focused on conifers) have been increasingly conducted in forests extending from subtropical to boreal regions (10, 12, 13). Earlier secondary growth resumption of conifers is now being reported in warmer years (14, 15), at lower latitudes (10, 15), and at lower altitudes (10, 16). Springtime (April to May) temperature was identified as the major factor triggering the onset of wood formation at mid to high latitudes in the Northern Hemisphere (10). However, chilling, forcing, and photoperiod are also assumed to play roles in regulating wood formation (14, 17). A chilling-influenced heat-sum model has also suggested that chilling and forcing determine the onset of wood formation of conifers in the Northern Hemisphere (18). Other exogenous factors, such as precipitation or moisture availability (19, 20), and endogenous factors, such as plant hormones (21, 22), also drive cambium onset and wood formation. A common shortcoming of these previous studies is that they have mainly focused on a single aspect of phenology without considering other exogenous factors, which were therefore poorly quantified. For example, Rossi et al. (10) did not assess the importance of factors such as photoperiod, moisture availability, forcing, and chilling. Similarly, Delpierre et al. (18) disregarded factors such as moisture availability or drought and did not quantify the importance of the studied factors in terms of their contributions to the onset of wood formation. Therefore, an integrated model is still lacking that would quantify the relationships between the onset of wood formation and several exogenous drivers to further our understanding of the mechanisms controlling wood production (18). Secondary growth determines stem radial growth and, in combination with primary growth, ultimately shapes tree morphology and function, thereby affecting forest productivity and carbon sequestration. A quantitative assessment of the main drivers triggering seasonal secondary growth resumption (mainly wood formation onset) is therefore critical for understanding global change and adaptive forest management.

Here, we quantified the exogenous drivers that contribute to secondary growth resumption of conifers in the Northern Hemisphere and their relative importance. To do this, we used a collection of extensive xylogenesis datasets of 826 individuals from 21 conifer species, distributed in 79 sites across subtropical, Mediterranean, temperate, and boreal biomes over the Northern Hemisphere latitudes ranging from 23 to 67°N. Given the divergence in life strategies among species (more vs. less prone to take risks) to cope with climate change and environmental stresses (e.g., drought and frost), we compared differences in these drivers across biomes and between early and late successional species (see more in *Materials and Methods*) to clarify the underlying mechanisms. We hypothesized that resumption of secondary growth or the onset of wood formation can be modeled as a function of photoperiod, temperature, moisture availability, forcing, and chilling, given that these external factors are critical for primary growth, and that primary and secondary growth are tightly connected (16).

## Results

### Geographical Patterns across Biomes.

**Fig. 1.**
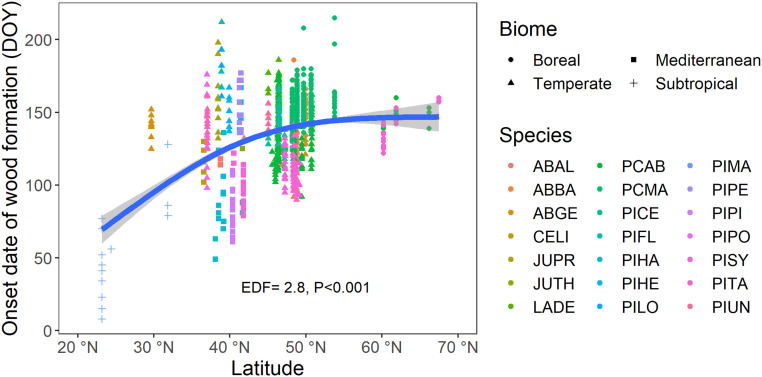
Variation in the onset date of wood formation (DOY) in relation to latitude as computed by a generalized additive model (GAM). The whole studied area was divided into the subtropical (cross), temperate (triangles), Mediterranean (squares), and boreal (dots) biomes. The species were reported with the following acronyms and classified into early (JUPR, JUTH, LADE, PIFL, PIHA, PIHE, PILO, PIMA, PIPE, PIPI, PIPO, PISY, PITA, PIUN) and late (ABAL, ABBA, ABGE, CELI, PCAB, PCMA, PICE) successional species types. Points (*n* = 2,030) represent individual trees from the 79 study sites included in this study. EDF, estimated degrees of freedom.

The date of onset of wood formation generally increased with latitude from subtropical to boreal biomes (Fig. 1). The earliest onset date of wood formation was observed on day of the year (DOY) 8 in the subtropical biome and the latest on DOY 215 in the boreal biome. A higher number of observations of wood formation onset was available in the dataset from mid to high latitudes (45 to 55°N), i.e., the temperate and boreal biomes, while fewer observations were from the Mediterranean (*n* = 191) and subtropical (*n* = 20) biomes. An average thermal range of −2.3 to 22.9 °C was observed across the study sites and over latitudes (*SI Appendix*, Table S1 and Figs. S1–S3). The DOY for onset of wood formation was delayed (i.e., the DOY was higher) with decreasing mean annual temperature of the site (MAT, computed for each site during the same year of wood formation) and with longer photoperiod at high latitudes (*SI Appendix*, Figs. S4 and S5).

### Drivers of Secondary Growth Resumption.

Mixed-effect models (model 1) showed that the onset date of wood formation for all of the studied tree species over the Northern Hemisphere could be modeled as a function of photoperiod, forcing, chilling, and the self-calibrating Palmer drought severity index (scPDSI), in addition to the random effects (Table 1). In this model, the marginal and conditional *R*^2^ values were 0.43 and 0.98, respectively. When replacing the photoperiod by the MAT, model 2 showed that the onset date of wood formation could be modeled as a function of both MAT, forcing, chilling, and scPDSI. When compared to the results of the trial models (*SI Appendix*, Table S2), the marginal *R*^2^ values increased to 0.84 and the conditional *R*^2^ values were 0.94. Overall, 34% of the additional variance was explained by forcing and chilling as well as by the scPDSI, compared to the model with the MAT alone. Of all of the models, model 2 was the most parsimonious and best described the quantitative relationships between the changes in the onset date of wood formation and exogenous factors (Table 1 and *SI Appendix*, Table S2).

**Table 1.** Statistics of model 1, including photoperiod, forcing, chilling, and scPDSI, and of model 2, including MAT, forcing, chilling, and scPDSI

	Model 1: photoperiod + forcing + chilling + scPDSI	Model 2: MAT + forcing + chilling + scPDSI				
		All (2,030)	Boreal (683)	Temperate (1,136)	Early (694)	Late (1,336)
Fixed effects						
Intercept	−160.1 (5.55)***	124.6 (1.70)***	127.3 (1.48)***	122.5 (2.58)***	129.7 (2.89)***	121.4 (2.15)***
Photoperiod, h	18.88 (0.35)***					
MAT, °C		−5.92 (0.13)***	−5.23 (0.20)***	−7.02 (0.30)***	−6.27 (0.17)***	−5.93 (0.19)***
Forcing, FU	0.05 (0.002)***	0.12 (0.002)***	0.11 (0.003)***	0.13 (0.003)***	0.12 (0.003)***	0.12 (0.003)***
Chilling, d	0.05 (0.01)***	0.23 (0.01)***	0.16 (0.02)***	0.28 (0.01)***	0.24 (0.02)***	0.25 (0.01)***
scPDSI	0.64 (0.08)***	1.09 (0.09)***	1.33 (0.10)***	0.89 (0.17)***	0.45 (0.19)*	1.26 (0.10)***
Random effects						
SD (site)	24.84	7.53	1.69	8.26	3.93	7.82
SD (species)	3.75	2.42	0.73	1.97	3.98	2.77
SD (residual)	4.62	5.85	4.81	6.17	6.28	5.64
Model fit						
Marginal *R*^2^	0.43	0.84	0.78	0.78	0.93	0.73
Conditional *R*^2^	0.98	0.94	0.81	0.93	0.96	0.91
AIC	12,510	13,246	4,137	7,557	4,645	8,635
BIC	12,555	13,291	4,173	7,597	4,682	8,677

Model 2 was the best model for explaining the onset of wood formation, and it was applied to all trees and to each group (boreal and temperate biomes; early and late successional species) separately. Values in the brackets on the top row are the number of observations. Note: For fixed effects, SEs are given in the brackets; ***P < 0.001; **P < 0.01; *P < 0.05; marginal *R*^2^ (fixed effects only); conditional *R*^2^ (both fixed and random effects). AIC, Akaike information criterion; BIC, Bayesian information criterion; MAT, mean annual temperature.

Consideration of the different biomes separately revealed that the same variables were related to the onset date of wood formation of trees from the boreal and temperate biomes as were found for all biomes combined (Table 1). Results for the Mediterranean biome showed that only MAT and forcing were significant (*SI Appendix*, Table S5); however, the results for the subtropical biome were unreliable and then excluded due to the low number of observations (*n* = 20). For both the early and late successional tree species, the same variables were significant, and 20% more variance was noted in the marginal *R*^2^ for the early species group than for the late species group (Table 1).

**Fig. 2.**
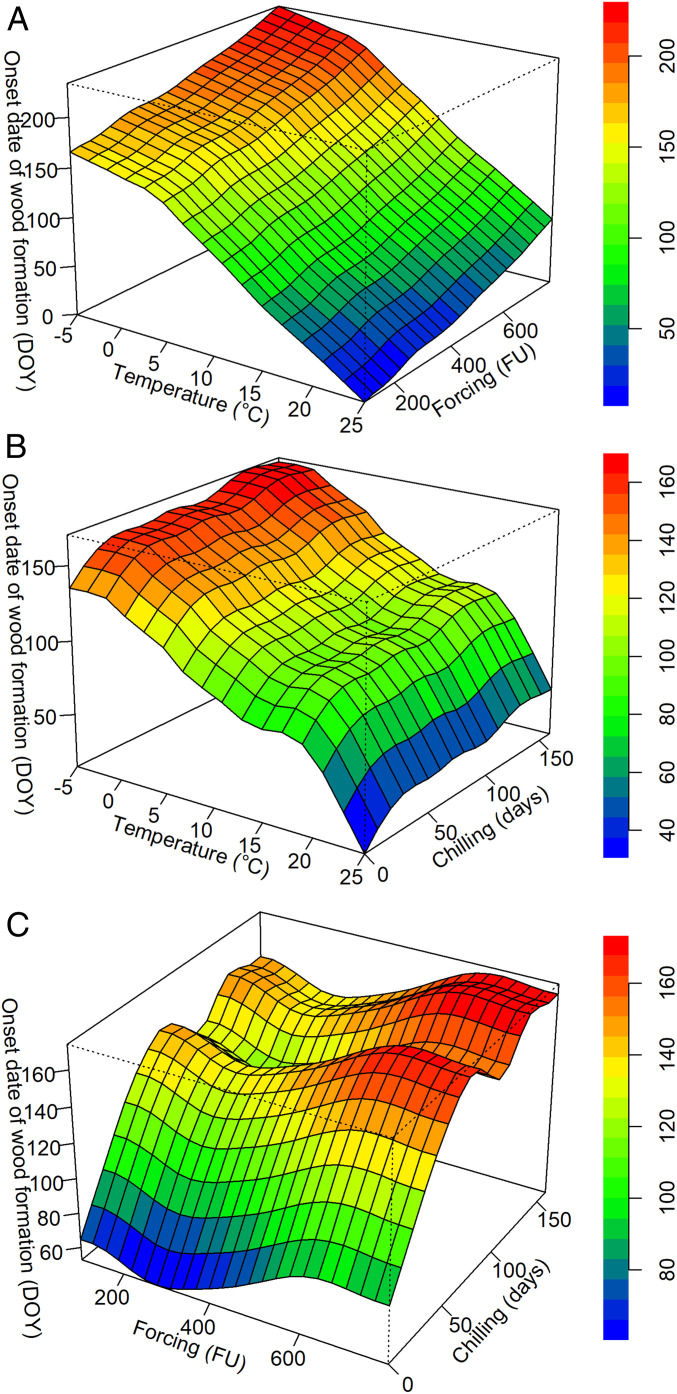
Changes in the onset date of wood formation (DOY) in relation to mean annual temperature (MAT) (abbreviated as “temperature”) and forcing (*A*), MAT and chilling (*B*), and forcing (FU) and chilling (*C*) (fitted by a GAM).

Along the temperature gradient, trees from warmer sites initiated xylem growth earlier and at a lower number of forcing and chilling days, while trees from colder sites started growth later at a higher number of forcing and chilling days (Fig. 2). The relationship (onset date of wood formation and MAT vs. scPDSI) was similar to the relationships with forcing and chilling and showed a flat variation in scPDSI (*SI Appendix*, Fig. S6). The relationship between the onset of wood formation and forcing and chilling showed an inverted “L” shape, indicating that the onset of wood formation mostly occurred at 20 to 150 chilling days and at 100 to 700 forcing units (Fig. 2). The relationships between the onset of wood formation and photoperiod vs. forcing and chilling also presented a similar “L” shape (*SI Appendix*, Fig. S7).

The variances explained by the different chilling thresholds ranged from 5.33 to 8.47% (*SI Appendix*, Table S6). In terms of the variance explained and biological meaning, the temperature range of −5 to 5 °C was the best threshold for the chilling calculation.

### Partition of Variance.

**Fig. 3.**
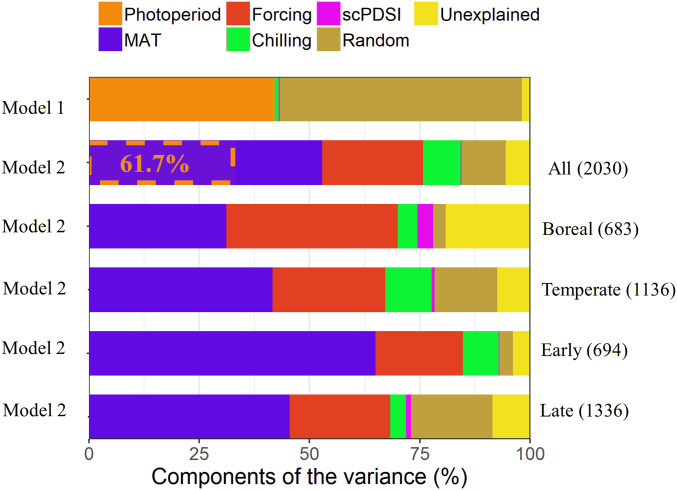
Variance partition of the studied variables. Fixed variables include photoperiod, forcing, chilling days, and scPDSI in model 1 (DOY ∼ photoperiod + forcing + chilling + scPDSI), and MAT (represented by mean annual temperature), forcing, chilling days, and scPDSI in model 2 (DOY ∼ MAT + forcing + chilling + scPDSI). Random variables include species and sites. Model 2 was applied to all trees and separately to trees within each group (the boreal and temperate biomes, and the early and late successional species). The values in the brackets represent the number of observations for the corresponding group. The value in the rectangles with dashed borders refers to the proportion of variance in MAT explained by photoperiod.

The photoperiod explained 42% of the variance in the onset of wood formation in model 1 (Fig. 3), when forcing, chilling, and scPDSI were added. This value was lower than the value obtained in the trial model using the photoperiod alone as the predictor (46%) (*SI Appendix*, Table S2). When photoperiod was replaced by the MAT, more variance in the onset of wood formation was explained in model 2 (84%) compared to model 1 (43%) (Fig. 3 and Table 1) and the trail model using MAT alone as the predictor (*SI Appendix*, Table S2).

When model 2 was applied to the geographical and tree species subgroups, the MAT accounted for 31% and 42% of the variance in the boreal and temperate biomes, and 65% and 46% of the variance in the early and late successional species, respectively (Fig. 3). Forcing was another important variable that contributed to the onset of wood formation, with 39%, 26%, and 23%, of the variance explained in the boreal, temperate, and all biomes, respectively. In addition, 20% and 23% of the variance was explained in the early and late successional species, respectively. Chilling accounted for a lower share of the variance, with 4.5%, 10.5%, and 8.5% in the boreal, temperate, and all biomes, respectively, as well as 8% and 3.6% in the early and late successional species, respectively. The scPDSI explained the least variance for the boreal (3.5%), temperate (0.6%), and all (0.2%) biomes, as well as for the early (0.0%) and late (1.1%) successional species. The random effects of site and species together accounted for 3%, 14%, and 10% of the variance in the boreal, temperate, and all groups, respectively, as well as 3% and 18.5% of the variance for the early and late successional species types, respectively. Unexplained variance ranged from 4 to 19% in all of the study groups. Across different biomes, trees tended to require higher forcing in the boreal than in the temperate biome. Between the life strategies, 3% more forcing days were required for the late successional species than for the early successional species. Chilling requirements for the temperate biome and the early successional species were double of the boreal and the late successional species, respectively.

## Discussion

The availability of a unique collection of extensive datasets of wood formation of 21 coniferous species over the Northern Hemisphere allowed the quantitative modeling of the onset of wood formation as a function of MAT, forcing, and chilling, as well as moisture availability. These data also enabled an evaluation of the relative importance of these factors as predictors of the onset of wood formation. We found that photoperiod was a key driver for the onset of wood formation of Northern Hemisphere conifers. Furthermore, photoperiod tightly interacted with MAT, as shown by the higher percentage of variance (61.7%) in MAT that was explained by photoperiod alone. Taken together, therefore, the onset of wood formation in conifers over the Northern Hemisphere was driven primarily by photoperiod and MAT or by their interaction, followed by forcing and chilling, and then by moisture availability. Photoperiod played a key role in regulating the onset of secondary growth (i.e., wood formation), contrary to its recognized, but often unquantified, role in affecting the springtime phenology of primary growth, such as leaf unfolding and budburst (4). Another important finding was that forcing played a more important role than chilling in initiating the onset of wood formation.

### Drivers of the Onset of Wood Formation.

#### Photoperiod.

Photoperiod plays a role in controlling phenology in many tree species (23), including autumn phenology, such as growth cessation and bud set preceding dormancy induced by short days (24, 25), and springtime phenology phases, such as budburst and leaf unfolding (4, 9, 26). However, how and to what extent photoperiod may affect the springtime phenology of primary growth are not clear and still under debate (4). Previous studies have reported that photoperiod effects may vary across species due to interactions with confounding factors, such as tree age (27) and successional niche (28). Therefore, a role for photoperiod has not been excluded but has not yet been quantified (4). The molecular mechanisms underlying photoperiodic influences are becoming better understood, as thoroughly reviewed by Jackson (24) and Singh et al. (25). However, the molecular mechanisms that drive growth cessation and bud set in autumn, prior to dormancy, are likely to differ from those driving dormancy or those triggering the springtime phenology of primary meristem growth after dormancy break. For instance, a recent study on hybrid aspen trees demonstrated that photoperiodic regulation of dormancy is mechanistically distinct from autumnal growth cessation (29).

Unlike the effect of photoperiod on primary meristems (23, 30), photoperiodic effects on secondary growth are less well explored. In the present study, we found that photoperiod was the key driver for the onset of wood formation and that it functioned through a strong interaction with the MAT. This finding is consistent with the results reported by Cuny et al. (31) who found that xylem growth had a stronger relationship with photoperiod than with light radiation intensity, soil water content, or temperature.

The photoperiodic signal controlling seasonal growth dynamics in trees is perceived by the leaves (24). Plants are thereby able to “measure” time (daylength) by an internal time-keeping mechanism, the circadian clock (24, 32). Therefore, the first mechanism introduced to explain how photoperiod perception by the foliage played this dominant role in wood formation in the tree stem is that the circadian clock differentially regulates systems of phytohormones, such as auxin (indole-acetic acid) and cytokinins (32, 33). These hormones are exported from source tissues (mainly apical meristems and actively growing leaves) and serve as mobile signals that regulate cambial cell division and xylem development (34, 35). Carbohydrate availability and signaling also have strong associations with cell division and expansion, because photosynthetic products act both as metabolic energy sources or carbon skeleton sources and as signaling molecules that direct gene expression through conserved signaling pathways to regulate plant growth and development (36). The mechanisms behind carbohydrate control of wood formation remain unclear. Future research on the molecular basis of wood formation will hopefully provide a better understanding of the interactions between photoperiodic pathways and other pathways and networks that regulate wood formation (32).

#### Air temperature.

We found that MAT was the second most important variable driving the onset of wood formation, based on the relative importance of the variances explained by MAT. MAT integrates the effect of local site conditions or average thermal conditions, including local climate, insolation (4), and landscape topology (slope and coordinates). MAT is a useful proxy for the driving forces of plant growth in terrestrial ecosystems (4, 10). For example, the simulated treeline position at a global scale was better predicted by a climate-driven model that considered temperature rather than latitude and altitude (37). MAT interacts with photoperiod in determining the timing of growth resumption (9, 23). However, the mechanism by which low temperature in early spring is sensed by trees and how low temperature interacts with photoperiod remain to be established (25). In addition, the effect of soil temperature on wood formation is not yet clear, due to the lack of soil temperature data. Our findings suggest that soil temperature might be far less important than the MAT, given the low unexplained variance in model 2 (6%).

#### Forcing and chilling.

Forcing and chilling were ranked as the third and fourth most important variables triggering the onset of wood formation, with a higher contribution for forcing (23%) than for chilling (8.5%). Forcing and chilling reflect the dual roles of temperature in the timing of springtime phenology of primary meristems (4, 7, 8). Cold temperatures in late autumn and winter (chilling) are required to break bud dormancy (the endodormancy phase), while warmer temperatures in springtime (forcing) are required to promote bud growth after dormancy has broken (ecodormancy phase) (7, 8). However, similar dual roles for temperature in triggering the onset of secondary meristem growth have only rarely been reported. Here, we present evidence for a role for both forcing and chilling, but we also showed that forcing plays a much more important role than chilling. Chilling temperatures may promote the accumulation of soluble sugars for frost protection (e.g., sucrose converted from starch) (38). A high sugar level may be retained until springtime de-hardening and could sustain cell production under favorable springtime forcing temperatures (18).

Our results suggest that a temperature range of −5 to 5 °C is the best threshold for chilling calculations. This chilling threshold is consistent with several previous studies (39, 40), but it differs from the most commonly used threshold of 0 to 5 °C (7). This difference suggests that the chilling requirement may also differ for primary and secondary growth types, and among species, provenances, and even among individuals (7). Previous studies on phenological models have also reported complex interactions between photoperiod and chilling and/or forcing (41, 42). By contrast, our empirical data did not show these interactive effects (*SI Appendix*, Fig. S7).

#### Moisture availability.

The scPDSI in the month prior to the onset of wood formation has a significant but low contribution (0.2%) to the onset, suggesting that moisture availability plays only a marginal role in triggering the onset of wood formation of the Northern Hemisphere conifers. Our finding is consistent with studies from dry environments, such as the Mediterranean Basin, the northeastern Tibetan Plateau, and Nevada, United States, where springtime moisture availability was critical for the onset of wood formation (19, 20, 43). This finding can be explained by the springtime rehydration period, which is an important phenomenon observed in springtime in boreal forests and occurs and lasts for about 40 d before wood formation onset (44). Springtime rehydration can therefore guarantee that trees recover an adequate water balance after a considerable water loss in the winter (45). This would sustain cell division and expansion because xylem cell expansion is a turgor-driven process that relies on cellular water uptake and solute accumulation (46).

### Variability across Biomes.

Comparison of the boreal and temperate biomes revealed that forcing played a more important role than MAT in initiating the onset of wood formation in the boreal biome, with less chilling requirements, given their similar variances explained by these three factors. These results might suggest that, despite the warming-induced chilling reduction reported for phenological phases such as leaf unfolding (4), both temperate and boreal trees still receive sufficient winter chilling to trigger the onset of wood formation. Consequently, warming may continue to induce an early onset of conifer wood formation. These results also suggest the possibility of a compensation mechanism between MAT and forcing and chilling. In other words, in the boreal biome, the low MAT means that more forcing but less chilling is needed. However, under the projected climate warming scenarios, boreal conifers will require less forcing but more chilling to trigger wood formation because of the higher MAT, which will create conditions more similar to the temperate biome. This also indicates that conifers in the Northern Hemisphere can adapt well to climate warming through phenological and physiological adjustments (high plasticity), although their mechanisms of genetic adaptation are not known.

Comparison of the Mediterranean biomes to the temperate and boreal biomes revealed that MAT and forcing were significant factors in the Mediterranean biomes but explained less variance. These findings suggested that MAT and forcing also played an important role in triggering the onset of wood formation (43). However, the chilling and moisture availability findings did not reach statistical significance. The reasons for this might be an insufficient number of observations per species and site in the Mediterranean biome, given the high variance accounted for by the random effects of species and sites.

### Early vs. Late Successional Species.

MAT contributed much more, and forcing less, to the onset of wood formation in the early successional species than in the late ones. This suggests that early successional conifers may benefit more than late successional species in terms of the effects of climate warming on the onset of wood formation. The early species have continuous flushes of new buds and leaves, whereas the late ones bear predetermined flushes. Therefore, the loss of the first flush in the event of an extreme climate could be compensated in the early species but not in the late species. For the same reason, increases in the frequency of springtime frosts as a result of climate warming (47) would be also less detrimental to the early than to the late species.

The forcing results are also consistent with previous studies showing a higher forcing requirement for late successional than for early successional species (30, 42). In addition, the MAT makes a greater contribution to the early successional species, suggesting a greater responsiveness of photoperiod in the early than in the late successional species, given the high dependence of MAT on photoperiod (photoperiod explains 61.7% of MAT variance). This finding is also consistent with several previous studies (30, 40, 42), but it contrasts with others (28). This discordance might reflect differences between the mechanisms driving secondary growth in the mature coniferous trees considered in our study vs. primary growth (twigs) in seedlings or young trees commonly used in previous studies (28, 30). Our finding of double chilling requirements in the early successional species vs. the late successional species contrasts with the results of previous studies showing that chilling requirements were more pronounced for late successional species (28, 42). The reasons underlying these differences merit further investigation.

## Conclusions

Global forests adapt to climate change through various mechanisms, including phenological changes. The challenge for global change scientists is to understand how forest trees respond and adapt to the ongoing warming through modulations of the springtime onset of growth. We used an extensive collection of unique cellular-level datasets of xylogenesis to provide a quantitative demonstration that the onset of wood formation in conifers of the Northern Hemisphere is primarily driven by photoperiod and MAT or their interactions, followed by forcing and chilling and then by moisture availability. This study is an attempt to integrate multiple exogenous factors that can affect the onset of wood formation in gymnosperms from subtropical to boreal biomes and across the Northern Hemisphere with the aim of elucidating the underlying mechanisms. Our results provide unique and insightful evidence supporting the regulation of wood formation of conifers by exogenous factors that can be incorporated into state-of-the-art Earth system models to improve the predictions of terrestrial carbon, water, and energy cycle changes under global change scenarios. Future studies that determine how exogenous factors regulate the other phases of wood formation may generate a deeper understanding of acclimation mechanisms in forests and trees.

## Materials and Methods

### Field Experiments and Sample Collection.

All of the experiments established for the study were based on criteria or procedures applied either in the field or laboratory, as described below. The date of onset of wood formation was determined by monitoring several healthy dominant vigorous trees of each representative tree species, ranging from 1 to 55 trees among all sites, throughout the growing seasons of 1998 to 2016 according to local climate conditions (*SI Appendix*, Table S1). Microcores of the stems were collected weekly (90%), or occasionally biweekly, at breast height (1.3 ± 0.3 m) using surgical bone-sampling needles or a Trephor tool. The microcores contained mature and developing xylem of the current year, the cambial zone, and the adjacent noncollapsed phloem, as well as at least the previous complete xylem tree ring. The microcores were fixed in solutions of propionic or acetic acid mixed with formaldehyde and stored in ethanol–water at 5 °C. In total, data were collected from 826 individuals of 21 conifers distributed across 79 sites that covered boreal, temperate, Mediterranean, and subtropical biomes in North America, Europe, and Asia. The sites were distributed over latitudes from 23°11′ N to 67°30′ N and at elevations ranging from 23 to 3,850 m above sea level (*SI Appendix*, Fig. S1 and Table S1). The investigated species, according to the literature, could be defined as early or late successional species. Early successional species are the shade-intolerant pioneers colonizing light-rich microhabitats or forest gaps created by disturbances, and able to grow vigorously and regenerate quickly on poor soil conditions and harsh environments. Late successional species are shade-tolerant, able to survive in the understory, dominating the mature or climax forests after the replacement of the pioneer species, and characterized by slow growth rate. The early successional species were *Juniperus przewalskii* Kom., *Juniperus thurifera* L., *Larix decidua* Mill., *Pinus flexilis* James., *Pinus halepensis* Mill., *Pinus heldreichii* Christ., *Pinus longaeva* Bailey, *Pinus massoniana* Lamb., *Pinus peuce* Griseb., *Pinus pinaster* Ait., *Pinus ponderosa* Douglas ex Lawson, *Pinus sylvestris* L., *Pinus tabulaeformis* Carr., and *Pinus uncinata* Mill. Ex Mirb. The late successional species were *Abies alba* Mill., *Abies balsamea* (L.) Mill., *Abies georgei* (var. smithii), *Cedrus libani* A. Rich, *Picea abies* L. Karst, *Picea mariana* (Mill.) B.S.P., and *Pinus cembra* L.

The microcores were dehydrated with successive immersions in ethanol and D-limonene, and then embedded in paraffin or glycol methacrylate (an exception was the samples from Switzerland, which were not embedded). The microcores were cut with rotary or sledge microtomes to obtain transverse sections of 10- to 30-µm thickness. The sections were stained with cresyl violet acetate or with a mixture of safranin and astra/Alcian blue, and then examined by light microscopy (bright-field and polarized light).

### Data of Onset of Wood Formation.

Cambial initials of the vascular cambium, which is a secondary meristem (21), divide both outward and inward to produce phloem and xylem mother cells that, in turn, form new phloem and xylem tissues (22). The process of tracheid formation includes cellular enlargement, secondary cell wall thickening and lignification, and then programmed cell death, leading to the mature phase. In each sample, tracheids in various phases of differentiation were counted along one to three radial rows. Enlarging tracheids contained a thin primary wall and had a radial diameter of at least twice that of cambial cells, which presented a fusiform shape. Tracheids in the cell wall thickening phase were identified by the birefringence of their secondary cell walls under polarized light (48); this birefringence discriminated them from enlarging tracheids. Mature tracheids had a completely developed cell wall, and they did not contain protoplasm. The mean number of xylem tracheids in the enlargement phase was obtained for each sampling date. The date of the onset of wood (xylem) formation, represented as DOY, was defined for each tree, site, and year as the date of appearance of the first enlarging cell. In total, 2,030 records for the onset of wood formation from 826 trees were included (*SI Appendix*, Table S1).

### Climate Data and Photoperiod.

A meteorological station was installed at most sites to measure climate conditions. For the remaining sites, data from the nearest meteorological stations were downloaded from the National Oceanic and Atmospheric Administration (NOAA) (https://www.ncdc.noaa.gov/cdo-web/datatools/findstation) and used (*SI Appendix*, Table S1). Temperature and precipitation data were derived from sensors fixed 2 to 3 m above the ground in the forest gaps beside or close to the sampled trees. Temperature was recorded hourly or subhourly, and daily mean, minimum, and maximum temperatures were calculated. Precipitation was recorded daily. Occasionally, a few missing or abnormal daily values were also obtained from estimates from the nearby weather stations from NOAA.

Rossi et al. (10) performed multiple comparisons of the effect of the current-year temperature over daily, weekly, monthly, and annual scales on wood formation and confirmed the MAT of the sites as one common and meaningful variable that describes xylem dynamics over the Northern Hemisphere. Thus, for our global modeling analysis, we computed the MAT from each site per year to represent the local climate of the sites in the studied regions and the average thermal conditions of that year for wood formation (10).

Soil moisture is also critical for triggering the onset of wood formation in semiarid and arid environments (19, 20), so we calculated total precipitation between January 1 and the onset of wood formation, as well as annual total precipitation. Two commonly used drought indices, the scPDSI and the standardized precipitation–evapotranspiration index (SPEI), were also applied to examine the effect of drought or moisture availability on the onset of wood formation. The scPDSI data with a spatial resolution of 0.5° were obtained from CRU scPDSI 4.03 (49). The SPEI values at 1-, 3-, and 6-mo scales, with a spatial resolution of 0.5°, were acquired from SPEIbase v.2.5 (50). For further modeling analysis, we obtained both scPDSI and SPEI from January to June, or combinations over multiple months, as well as for the month before the onset of wood formation.

Photoperiod, or daylength, was calculated using the R package “insol” (https://meteoexploration.com/R/insol/). Photoperiod does not change from year to year for a fixed date per site, but it varies with the onset date of wood formation of individual trees.

### Chilling and Forcing.

Chilling temperatures, which enable plants to release from the dormant state (51), were calculated at each site as the number of days in which the daily mean temperature was between −5 and 5 °C, based on the time span commonly used from November 1 in the previous year to the onset date of wood formation (4). Alternatively, chilling was calculated for trial modeling analysis when daily mean temperature was between 0 and 5 °C, between −5 and 0 °C, or between −10 and 0 °C, for the same reference period.

Forcing was computed using a sigmoid function of the average daily air temperature, as follows:

$$\mathrm{FU}=\overset{t_{d}}{\underset{t_{0}}{\sum }}D_{\mathrm{FU}}\;\text{if}\;T_{t}>T_{th}\;\text{where}\;D_{\mathrm{FU}}=\frac{28.4}{1+e^{-0.185\left(T_{t}-18.4\right)}},$$

where FU is the forcing unit for the onset of wood formation, *D*_FU_ is the daily forcing unit, *t*_0_ is the starting date for forcing accumulation (assumed here to be January 1, as commonly used) (4), *t*_*d*_ is the date of onset of wood formation, *T*_*t*_ is the mean daily air temperature, and *T*_*th*_ is the threshold temperature for forcing accumulation. Temperatures above 5 °C normally contribute to forcing in temperate regions, so those temperatures were used as the *T*_*th*_ (13) (see more in *SI Appendix*, *Methods S1*).

### Statistical Analyses.

Generalized additive models (GAMs) were used to describe the general trend in the date of onset of wood formation against MAT and latitude (*SI Appendix*, *Methods S2*). To assess the potential difference across biomes and between life strategies, the 79 sites included in the study were divided into subtropical, Mediterranean, temperate, and boreal biomes according to climate conditions of the sites, and the tree species were separated into early and late successional species.

Given the interaction effect between photoperiod and temperature reported previously (4, 9), and to detect their collinearity, we performed linear regressions between photoperiod and MAT to quantify their interactive relationship. Photoperiod explained 61.7% of total variance of the MAT (*SI Appendix*, Fig. S2). To separate their respective roles in triggering wood formation onset, linear mixed-effects models (LMMs) were used to test the effect of exogenous variables (photoperiod, MAT, moisture availability [i.e., precipitation, SPEI, and scPDSI], forcing, and chilling) on the onset of wood formation, while including species and site as random effects. Our LMMs were built at the site/species level instead of the individual tree level because our study interests focus on the biomes and life strategies in the Northern Hemisphere, rather than on the intersite and between-species differences reported previously. We used a conventional mixed-effects model building process (52) starting from a null model and then gradually extended to the higher levels to fit our hierarchical data. In the LMMs, we calculated the marginal and conditional *R*^2^ values, which account for fixed and fixed plus random effects, respectively. The minimum Akaike information criterion (AIC) and Bayesian information criterion (BIC) were used to select the best model (53, 54).

The preliminary LMM modeling analyses showed that photoperiod or MAT alone was a significant variable driving the onset of wood formation (*SI Appendix*, *Methods S3* and Table S2). The results also showed that annual total precipitation was not a significant variable, although total precipitation between January 1 and the onset date of wood formation was significant, as the marginal *R*^2^ (0.82) was lower than without it (0.84) (Table 1 and *SI Appendix*, Table S3). The variance partition also showed that total precipitation between January 1 and the onset date of wood formation had a low contribution to wood formation onset (*SI Appendix*, Table S3). Among all of the variables tested involving moisture availability, the scPDSI in the month before the onset of wood formation had the highest marginal *R*^2^ and was included in the final model. The results of the SPEI were similar to those for the scPDSI and are reported in *SI Appendix*, Table S4.

The LMM (model 1) was then used to model the changes in the onset date of wood formation with photoperiod, forcing, chilling, and scPDSI. The interactive terms among the four fixed-effect variables had variance inflation factors (VIFs) >5 and were therefore excluded from model 1 to avoid collinearity.Model 1:

$$D_{ijk}=\alpha +\beta_{1}P_{ijk}+\beta_{2}F_{ijk}+\beta_{3}C_{ijk}+\beta_{4}PDSI_{ijk}+a_{i}+b_{j}+\varepsilon_{ijk},$$

where *D*_*ijk*_ is the date of onset of wood formation of species *i* at site *j* in year *k*, and *P*_*ijk*_, *F*_*ijk*_, *C*_*ijk*_, and *PDSI*_*ijk*_, respectively, represent the photoperiod, forcing, chilling, and scPDSI corresponding to *D*_*ijk*_; *α* is the intercept; *β*_1_, *β*_2_, *β*_3_, and *β*_4_ are the slopes; *a*_*i*_ and *b*_*j*_ are, respectively, the random effect of the species *i* and site *j*; and *ε*_*ijk*_ is the error term.

A second LMM (model 2) was used to model changes in the onset date of wood formation driven by MAT, chilling, forcing, and scPDSI, without the photoperiod. The interactive terms among the four fixed-effect variables mentioned above were excluded from this model to avoid collinearity (VIF > 5):Model 2:

$$D_{ijk}=\alpha +\beta_{1}T_{ijk}+\beta_{2}F_{ijk}+\beta_{3}C_{ijk}+\beta_{4}PDSI_{ijk}+a_{i}+b_{j}+\varepsilon_{ijk},$$

where *T*_*ijk*_ represents the MAT corresponding to *D*_*ijk*_; the other parameters are the same as in model 1.

Finally, LMM was also tested by adding altitude as an independent variable to model 2. However, the altitude was not a significant variable and so was excluded from the final model. Therefore, model 2 was considered the best model to describe the relationships between wood formation onset and exogenous factors in terms of parsimony by minimizing AIC and BIC.

Model 2 was then used to predict the changes in the onset date of wood formation for each biome (i.e., subtropical, Mediterranean, temperate, and boreal) and life strategy as a function of MAT, chilling, forcing, and scPDSI, including species and site as random effects.

For each LMM, the contribution of each independent variable to the dependent variable was calculated by performing a decomposition of variance to partition the variances explained by each fixed variable, random variable, and unexplained component (55) (see https://github.com/mastoffel/partR2).

All data analyses were conducted in R (56).

## Supplementary Material

Supplementary File

Supplementary File

Supplementary File

## Data Availability

The data that support the findings of this study are provided in Dataset S1. Readers can access the full code in Code S1.
