# Supplementary material for: Photoperiod and temperature as dominant environmental drivers triggering secondary growth resumption in Northern Hemisphere conifers
Source: Proc Natl Acad Sci U S A. 2020 Aug 5;117(34):20645–52. doi: 10.1073/pnas.2007058117 (PMC7456155; doi:10.1073/pnas.2007058117)
Supplement: Supplementary File [file pnas.2007058117.sapp01.pdf]

www.pnas.org

Supplementary Information for

Photoperiod and temperature as dominant environmental drivers triggering secondary growth resumption in Northern Hemisphere conifers

Jian-Guo Huang[a, b, 1, 2], Qianqian Ma[a, b, 1], Sergio Rossi[a, c], Franco Biondi[d], Annie Deslauriers[c], Patrick Fonti[e], Eryuan Liang[f], Harri Mäkinen[g], Walter Oberhuber[h], Cyrille B.K. Rathgeber[i], Roberto Tognetti[j], Václav Treml[k], Bao Yang[l], Jiao-Lin Zhang[m,b], Serena Antonucci[j], Yves Bergeron[n], J. Julio Camarero[o], Filipe Campelo[p], Katarina Čufar[q], Henri E. Cuny[r], Martin De Luis[s], Alessio Giovannelli[t], Jožica Gričar[u], Andreas Gruber[h], Vladimír Gryc[v], Aylin Güney[w,x], Xiali Guo[a, b], Wei Huang[y], Tuula Jyske[g], Jakub Kašpar[k], Gregory King[e, z], Cornelia Krause[c], Audrey Lemay[c], Feng Liu[aa,b], Fabio Lombardi[bb], Edurne Martinez del Castillo[s], Hubert Morin[c], Cristina Nabais[p], Pekka Nöjd[g], Richard L. Peters[e, cc], Peter Prislan[q], Antonio Saracino[dd], Irene Swidrak[h], Hanuš Vavrčík[v], Joana Vieira[p], Biyun Yu[a, b], Shaokang Zhang[a, b], Qiao Zeng[ee], Yalin Zhang[a], and Emanuele Ziaco[d]

Corresponding author: Jian-Guo Huang
Email: huangjg@scbg.ac.cn

This PDF file includes:

Supplementary text
Methods S1 to S3
Texts S1 to S2
Figures S1 to S7
Tables S1 to S6

www.pnas.org/cgi/doi/10.1073/pnas.2007058117

Dataset S1

References

Other supplementary materials:

Code S1

Supplementary Information Text

Methods S1 to S3

S1: *Chilling and forcing*

Trees in temperate and cold ecosystems usually enter the dormancy state in later autumn or early winter after growth cessation and bud set in late summer or early autumn. The dormancy state involves the "rest" period (also called endodormancy phase) and the "quiescence" period (also called ecodormancy phase) (1). During the rest period, trees requires to be exposed to low chilling temperatures for a longer period (days to months) to break "rest" state. At the time of rest completion, the "quiescence" period is attained. During quiescence, the buds attain full ontogenetic competence, which is the full ability to respond to high forcing temperatures by anatomic development towards bud burst or cambial cell division (2). The chilling requirement is thus normally defined as the length of the period (days or hours) during which temperature remains within a specific range (3). Compared to the most effective temperature range of 0~5 ℃ usually calculated starting from November 1st of the previous year to the onset day of spring phenology of primary meristems reported previously (3), we found the temperature range of -5~5 ℃ is most effective for wood formation onset during our trial modeling analyses (*SI Appendix Table S6*). We therefore summarized the days when daily temperature was within this range, starting from November 1st of the previous year to the onset day of wood formation. The forcing requirement is normally defined as the length of period (days or hours) during which temperature remains above a specific threshold. The temperature threshold above 5 ℃ for the period starting from January 1st to the onset day of spring primary phenology was considered to be most effective and commonly used to calculate spring forcing requirement (2). We thus followed this threshold and the same reference period mentioned above for calculation of forcing requirement for wood formation onset.

S2: *Trends fitted by generalized additive models (GAMs)*

Generalized additive models (GAMs) were used to examine the general trend in mean annual temperature (MAT) and photoperiod of sites against latitude. Linear regression was performed to quantify the relationship between the onset date of wood formation and the photoperiod of the sites.

GAMs were also applied to examine the changes in the onset date of wood formation in relation to MAT and the scPDSI drought index, as well as the changes in the onset date of wood formation in relation to photoperiod, chilling, and forcing.

S3: *Preliminary analysis with linear mixed models (LMM)*

A linear mixed effects model (LMM), including species and site as random effects, was used to model the changes in the dates of onset of wood formation with photoperiod.

$$D_{ijk} = \alpha + \beta_1 P_{ijk} + a_i + b_j + \varepsilon_{ijk}$$

where D_{ijk} is the date of onset of wood formation of species i at site j in year k; P_{ijk} represents the photoperiod corresponding to D_{ijk}; α is the intercept; β_1 is the slope, and a_i and b_j, are, respectively, the random effects of the species i and site j; and ε_{ijk} is the error term.

In addition, LMM was also used to model the changes in the dates of onset of wood formation with MAT instead of photoperiod.

$$D_{ijk} = \alpha + \beta_1 T_{ijk} + a_i + b_j + \varepsilon_{ijk}$$

where T_{ijk} represents the MAT corresponding to D_{ijk}, while the other parameters are the same as described above.

Texts S1 to S2

S1: *General trend revealed by GAM*

The fitted generalized additive model (GAM) shows that the mean annual temperature (MAT) of the sites generally decreases toward higher latitudes (*SI Appendix, Fig. S3*), but the photoperiod of the sites increases toward higher latitudes (*SI Appendix, Fig. S5*). The

onset of wood formation was positively and linearly associated with photoperiod (R^2=0.68, $p<0.001$) (*SI Appendix, Fig. S5*).

The relationships between the onset of wood formation and MAT and scPDSI show a downward surface, where the onset of wood formation is negatively related with MAT but shows a flat variation in the scPDSI (*SI Appendix, Fig. S6*). The relationships between the onset of wood formation and photoperiod *vs.* forcing and chilling also presented an "L" shape. The "L" shape reflects less variability in days of the year (DOY; ca. 140–160) when photoperiod continues changing at high latitudes (*SI Appendix, Fig. S7*).

S2: *Photoperiod or MAT alone as a significant factor affecting wood formation onset*

Our LMM results show that the changes in the dates of wood formation onset in the Northern Hemisphere conifers can be modeled as a function of photoperiod alone, along with the random effects of site and species. The marginal R^2 and conditional R^2 are 0.46 and 0.98, respectively (*SI Appendix, Table S2*).

When photoperiod was replaced by the mean annual temperature (MAT), our LMM results show that the changes in dates of wood formation onset in the Northern Hemisphere conifers can be modeled as a function of MAT alone, along with the random effects of site and species. The marginal R^2 and conditional R^2 is 0.50 and 0.85, respectively (*SI Appendix, Table S2*).

Figures S1-S7:

Fig. S1. Location of the study sites across the Northern Hemisphere, according to altitude and biome type.

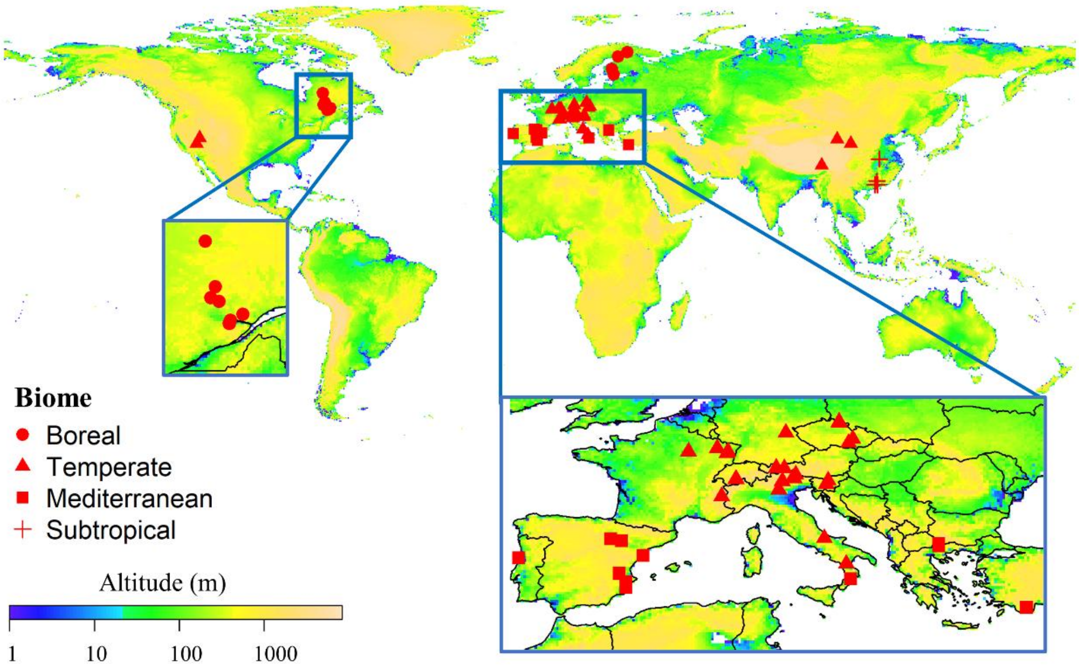

Fig. S2. Linear regression between photoperiod and the mean annual temperature (MAT) of the sites. The whole study area was divided into the subtropical (cross), temperate (triangles), Mediterranean (squares), and boreal (dots) biomes. The species were reported with the following acronyms and classified into early (JUPR, JUTH, LADE, PIFL, PIHA, PIHE, PILO, PIMA, PIPE, PIPI, PIPO, PISY, PITA, PIUN) and late (ABAL, ABBA, ABGE, CELI, PCAB, PCMA, PICE) successional species types. Points (n=2030) represent individual trees from the 79 study sites included in this study.

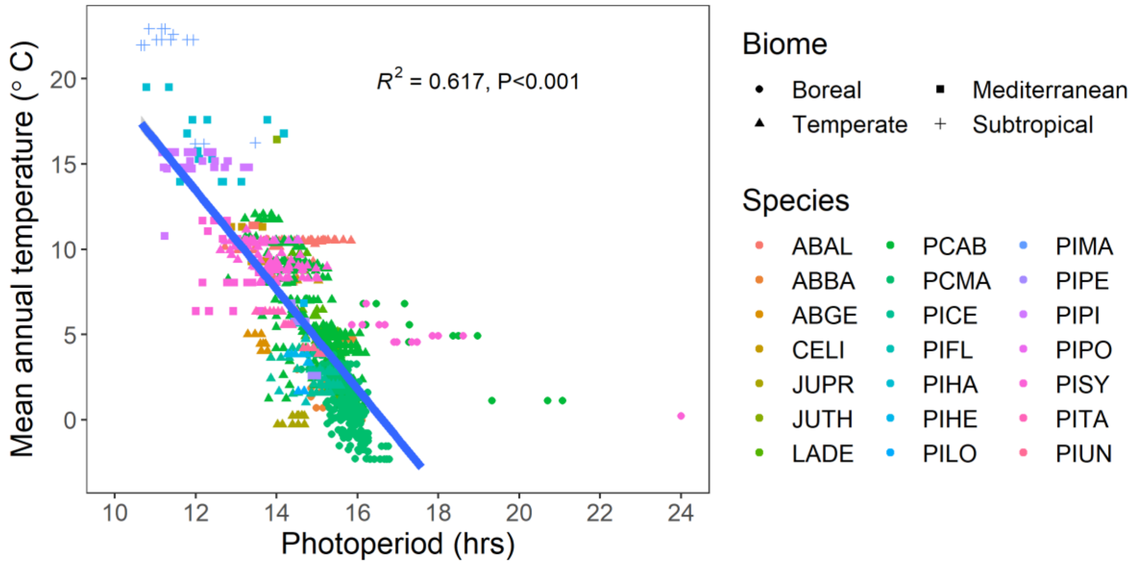

Fig. S3. Mean annual temperature (MAT) of the sites against latitudes (smoothed line resulted from generalized additive model GAM). The whole study area was divided into the subtropical (cross), temperate (triangles), Mediterranean (squares), and boreal (dots) biomes. The species were reported with the following acronyms and classified into early (JUPR, JUTH, LADE, PIFL, PIHA, PIHE, PILO, PIMA, PIPE, PIPI, PIPO, PISY, PITA, PIUN) and late (ABAL, ABBA, ABGE, CELI, PCAB, PCMA, PICE) successional species types. Points (n=2030) represent individual trees from the 79 study sites included in this study. EDF: estimated degree of freedom.

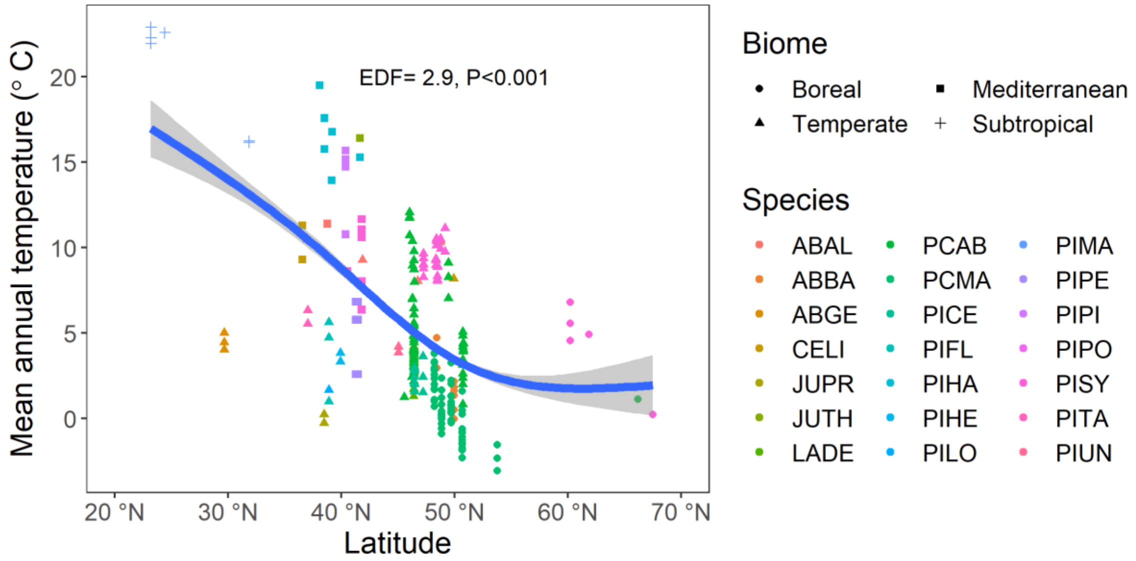

Fig. S4. Variation in the onset date of wood formation (DOY, day of the year) according to the mean annual temperature (MAT) of the sites (smoothed line resulted from generalized additive model GAM). The whole study area was divided into the subtropical (cross), temperate (triangles), Mediterranean (squares), and boreal (dots) biomes. The species were reported with the following acronyms and classified into early (JUPR, JUTH, LADE, PIFL, PIHA, PIHE, PILO, PIMA, PIPE, PIPI, PIPO, PISY, PITA, PIUN) and late (ABAL, ABBA, ABGE, CELI, PCAB, PCMA, PICE) successional species types. Points (n=2030) represent individual trees from the 79 study sites included in this study. EDF: estimated degree of freedom.

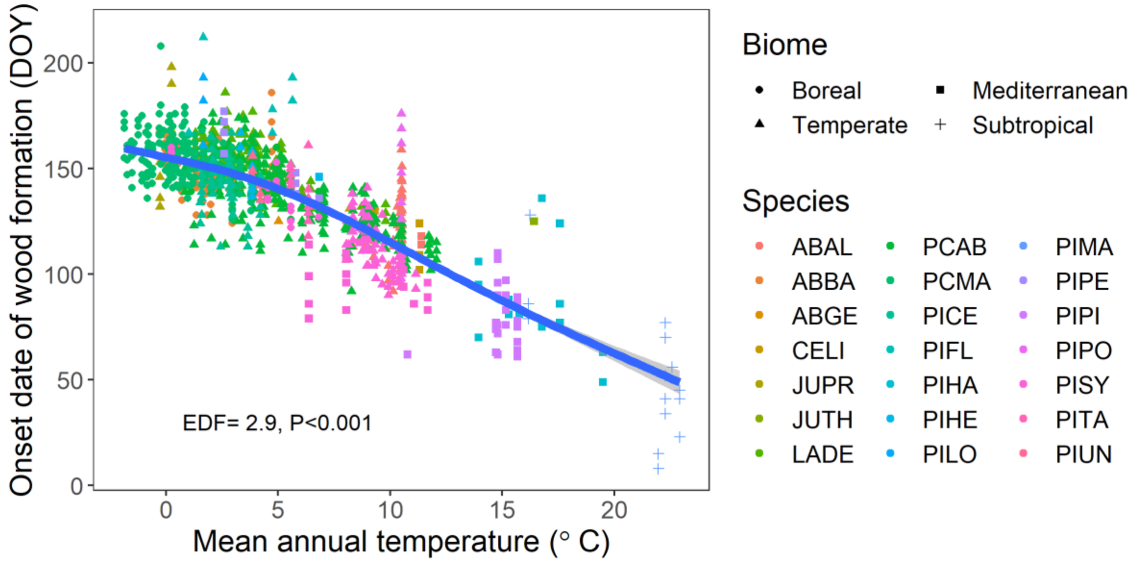

Fig. S5. Variation in the onset of wood formation (DOY, day of the year) according to the photoperiod of the sites (straight line fit from linear regression) (lower panel), and photoperiod varies with latitudes (upper panel). The whole study area was divided into the subtropical (cross), temperate (triangles), Mediterranean (squares), and boreal (dots) biomes. The species were reported with the following acronyms and classified into early (JUPR, JUTH, LADE, PIFL, PIHA, PIHE, PILO, PIMA, PIPE, PIPI, PIPO, PISY, PITA, PIUN) and late (ABAL, ABBA, ABGE, CELI, PCAB, PCMA, PICE) successional species types. Points (n=2030) represent individual trees from the 79 study sites included in this study. EDF: estimated degree of freedom.

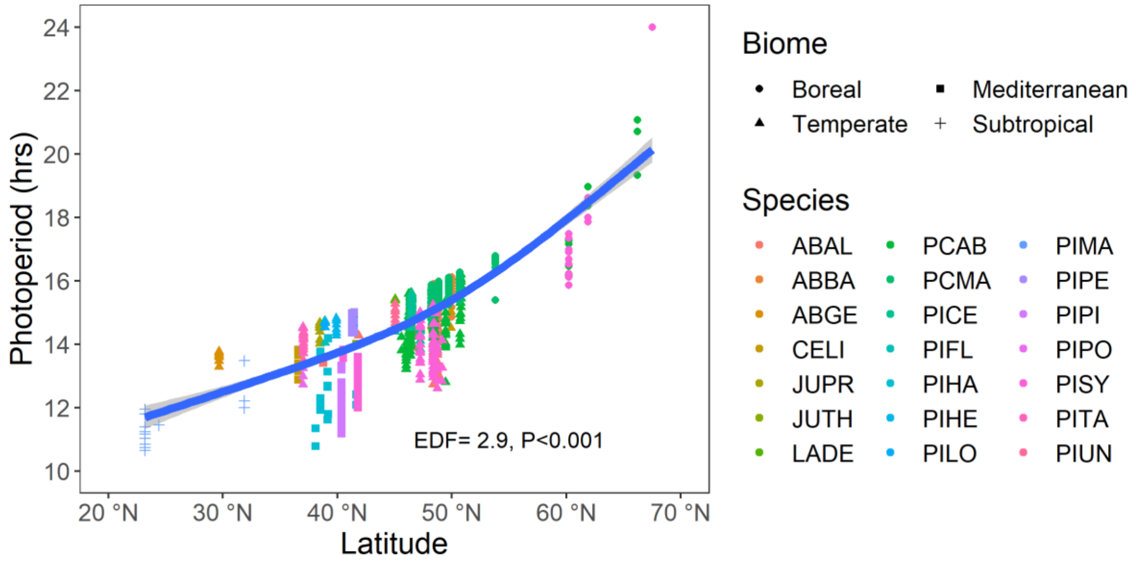

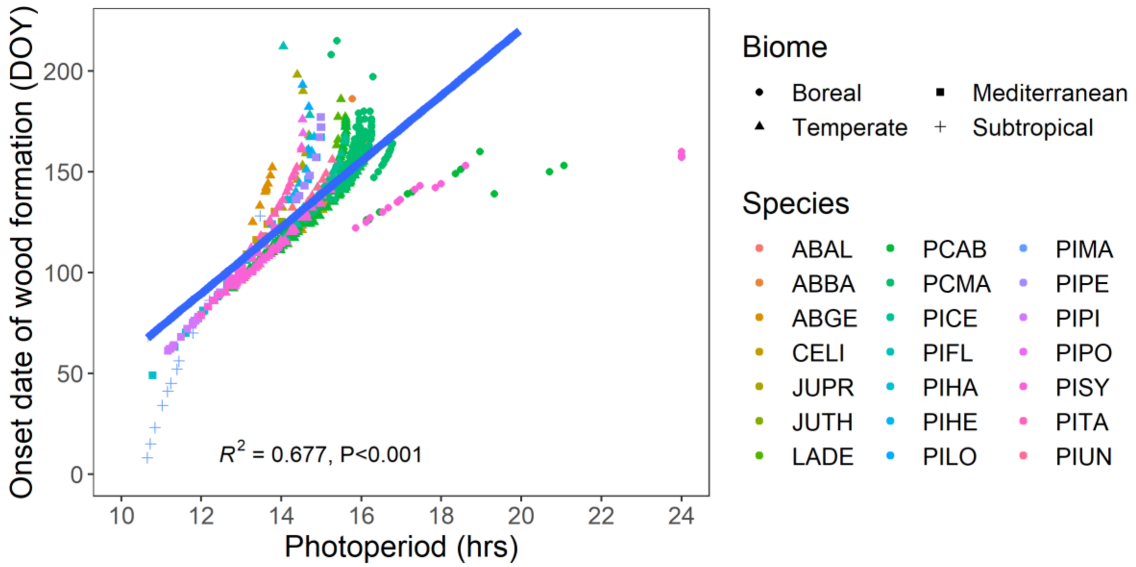

Fig. S6. Changes in the onset of wood formation (DOY, day of the year) in relation to MAT (mean annual temperature, abbreviated as "temperature" in the x-axis) and the scPDSI drought index (fitted by a generalized additive model).

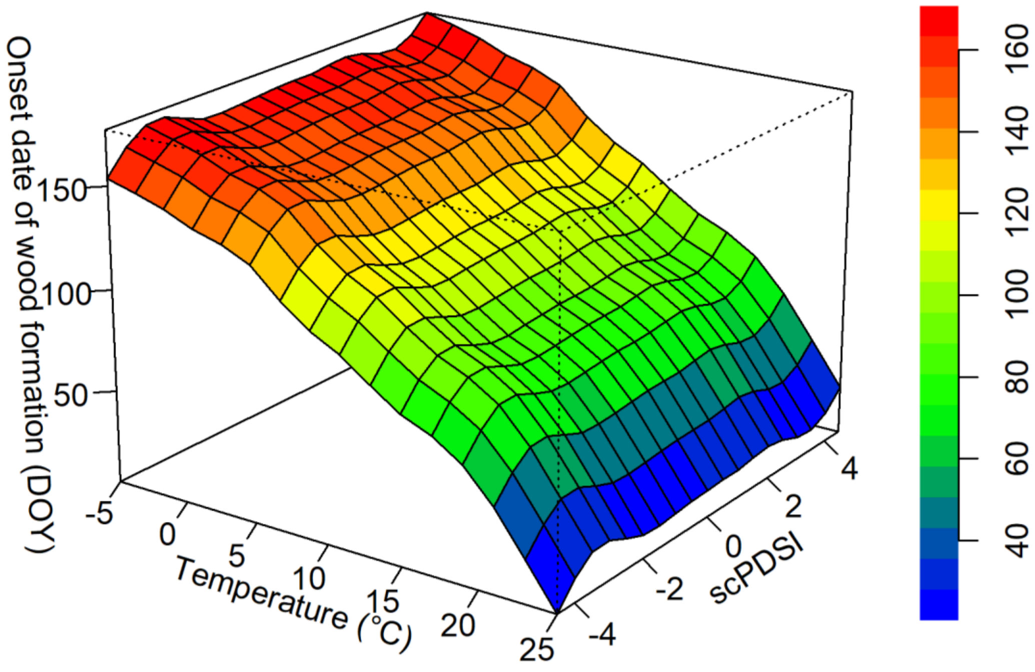

Fig. S7. Changes in the onset of wood formation (DOY, day of the year) in relation to photoperiod, chilling, and forcing (FU) days (fitted by a generalized additive model).

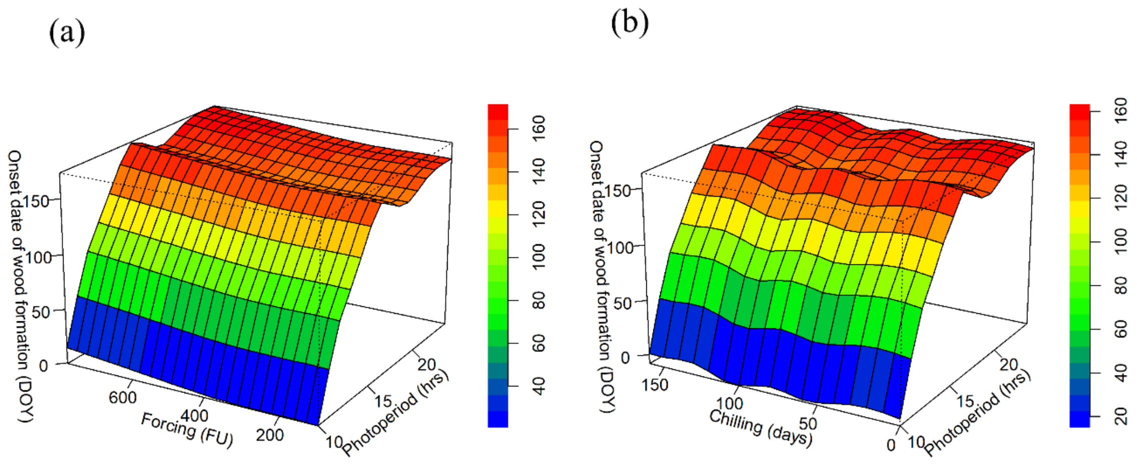

Table S1. Sites, species, and years included in the analysis. The species were reported with the following acronyms and classified into early (E) and late (L) successional species type: ABAL, *Abies alba*, L; ABBA, *Abies balsamea*, L; ABGE, *Abies georgei*, L; CELI, *Cedrus libani*, L; PCAB, *Picea abies*, L; PCMA, *Picea mariana*, L; PICE, *Pinus cembra*, L; JUPR, *Juniperus przewalskii*, E; JUTH, *Juniperus thurifera*, E; LADE, *Larix decidua*, E; PIFL, *Pinus flexilis*, E; PIHA, *Pinus halepensis*, E; PIHE, *Pinus heldreichii*, E; PILO, *Pinus longaeva*, E; PIMA, *Pinus massoniana*, E; PIPE, *Pinus peuce*, E; PIPI, *Pinus pinaster*, E; PIPO, *Pinus ponderosa*, E; PISY, *Pinus sylvestris*, E; PITA, *Pinus tabulaeformis*, E; PIUN, *Pinus uncinata*, E. The whole study area is divided into subtropical (S), temperate (T), Mediterranean (M), and boreal (B) biomes. Site temperature for each site is computed as the average of mean annual temperatures (MATs) across all years providing observations for the site. Sites with climate data obtained from nearby weather stations are indicated by *.

ID	Site	Biome	Latitude	Longitude	Altitude (m a.s.l.)	Study years	Species	Successional stage	Number of trees	Site temperature(℃)	References
DHS	CN-Dinghu Mountain	S	23°11'N	112°32'E	256	2015	PIMA	E	4	22.9	4
ZWY	CN-SCBG	S	23°11'N	113°22'E	23	2015-2016	PIMA	E	5	22.2	
SMT	CN-Shimentai	S	24°24'N	113°12'E	261	2015	PIMA	E	4	22.6	
SYG	CN-Sygera Mountain	T	29°39'N	94°42'E	3850	2007-2009	ABGE	L	5	4.5	5
JGS	CN-Jigong Mountain	S	31°51'N	114°5'E	811	2014-2015	PIMA	E	3	16.2	6
T2	TU-Cedar Research Forest	M	36°34'N	29°57'W	1960	2013	CELI	L	3	9.3	7
T4	TU-Cedar Research Forest	M	36°34'N	29°57'W	1055	2013	CELI	L	3	11.3	7
T1	TU-Cedar Research Forest	M	36°34'N	29°57'W	1665	2013	CELI	L	3	9.3	7
T3	TU-Cedar Research Forest	M	36°34'N	29°57'W	1355	2013	CELI	L	3	11.3	7
SHM	US-Sheep Range Montane	T	36°59'N	115°21'W	2320	2015-2016	PIPO	E	12	10.6	8
HSM	CN-Hasi Mountains	T	37°02'N	104°28'E	2456	2013-2014	PITA	E	9	5.9	9
GUA	ES-Guardamar del Segura	M	38°06'N	0°39'W	15	2005	PIHA	E	6	19.5	10
SDL	CN-Sidalong Forestry Station	T	38°27'N	99°56'E	3550	2013-2014	JUPR	E	9	-0.6	9
MAI	ES-Maimó	M	38°31'N	0°38'W	845	2004-2005	PIHA	E	10	16.9	11
SER	IT-Serra San Bruno	M	38°46'N	16°31'E	1008	2015	ABAL	L	5	11.4	12
SSM	US-Snake Range Montane	T	38°53'N	114°20'W	2810	2013-2014	PIFL	E	5	5.0	13
SSW	US-Snake Range Subalpine	T	38°54'N	114°18'W	3355	2013-2014	PIFL, PILO	E	10	1.6	13,14
JAR	ES	M	39°15'N	1°15'W	571	2005	PIHA	E	6	14.0	11
JAN	ES	M	39°19'N	1°15'W	850	2004	PIHA	E	2	16.8	11
POL	IT-Pollino	T	39°54'N	16°12'E	2053	2003-2004	PIHE	E	10	3.6	15

ID	Site	Biome	Latitude	Longitude	Altitude (m a.s.l.)	Study years	Species	Successional stage	Number of trees	Site temperature(℃)	References
TCH	PT	M	40°22'N	8°49'W	15	2010-2014	PIPI	E	22	14.7	16
VIL	ES-Villarroya de los Pinares	M	40°31'N	0°39'E	1690	2005	PISY	E	5	8.6	17
TDR	BG-Bunderitsa valley	M	41°26'N	23°25'E	1780	2012-2014	PIHE, PIPE	E	10	5.5	11
VRN	BG-Bunderitsa valley	M	41°27'N	23°15'E	1850	2012-2014	PIHE, PIPE	E	10	5.3	11
PEN	ES-Peñaflor	M	41°47'N	0°58'W	340	2006-2010	JUTH, PIHA	E	27	15.7	18
MYH	ES-Moncayo	M	41°47'N	0°43'W	1600	2011-2013	PISY	E	6	8.3	11
MYL*	ES	M	41°47'N	1°49'W	1600	2011-2012	PISY	E	6	11.4	11
PES	IT-Pescopennataro	T	41°52'N	14°30'E	1380	2015	ABAL	L	5	9.2	12
SUS	IT-Val di Susa	T	45°3'N	6°40'E	2030	2003-2004	LADE, PICE,PIUN	E,L,E	15	4.0	19
SAV*	IT-Lavazè	T	45°34'N	11°02'E	667	2010	PCAB	L	3	11	20
PAN	SI-Panska reka	T	46°0'N	14°40'E	400	2009-2012	PCAB	L	12	11.6	21
LAV	IT-Lavaze	T	46°13'N	11°18'E	1776	2010	PCAB	L	3	2.4	10
MEN	SI-Menina Planina	T	46°16'N	14°48'E	1200	2009-2012	PCAB	L	13	7.2	21
N22	CH-Lötschental-N22	T	46°22'52"N	7°46'22"E	2182	2007-2010	LADE	E	4	2.2	11, 22
N08	CH-Lötschental-N08	T	46°18'9"N	7°44'27"E	804	2008-2010	LADE, PCAB	E, L	8	9.3	11, 22
N13d	CH-Lötschental-N13d	T	46°23'30"N	7°45'40"E	1361	2007-2013	LADE, PCAB	E, L	8	5.5	11, 22
N13w	CH-Lötschental-N13w	T	46°23'36"N	7°45'50"E	1321	2013	LADE, PCAB	E, L	6	4.0	11, 22
N16	CH-Lötschental-N16	T	46°23'14"N	7°45'52"E	1634	2007-2010	LADE, PCAB	E, L	8	4.9	11, 22
N19	CH-Lötschental-N19	T	46°23'13"N	7°46'26"E	1961	2007-2010	LADE, PCAB	E, L	8	3.1	11, 22
S16	CH-Lötschental-S16	T	46°23'50"N	7°45'19"E	1670	2007-2013	LADE, PCAB	E, L	8	4.8	11, 22
S19	CH-Lötschental-S19	T	46°23'48"N	7°44'45"E	1928	2007-2013	LADE, PCAB	E, L	8	3.7	11, 22

ID	Site	Biome	Latitude	Longitude	Altitude (m a.s.l.)	Study years	Species	Successional stage	Number of trees	Site temperature(°C)	References
S22	CH-Lötschental-S22	T	46°23'59"N	7°44'33"E	2104	2007-2013	LADE	E	4	3.1	11,22
SVT	IT-San Vito di Cadore	T	46°26'N	12°13'E	1000	2003	PCAB	L	1	8.0	23
5T1	IT-Cinque Torri 1	T	46°27'N	12°8'E	2085	2001-2005	LADE, PCAB, PICE	E, L, L	23	2.4	24
5T3	IT-Cinque Torri 3	T	46°27'N	12°8'E	2085	2004-2005	LADE, PCAB, PICE	E, L, L	15	1.8	25
5T2	IT-Cinque Torri 2	T	46°28'N	12°8'E	2156	2002-2005	LADE, PCAB, PICE	E, L, L	12	2.3	24
BOR	IT-Borca di Cadore	T	46°44'N	12°19'E	1150	2015	ABAL	L	5	8.1	12
TIM	AT-Patscherkofel-timberline	T	47°12'N	11°27'E	1950	2007	PICE	L	6	3.7	26
TRE	AT-Patscherkofel-treeline	T	47°12'N	11°27'E	2110	2007	PICE	L	4	2.4	26
KRU	AT-Patscherkofel-krummholz	T	47°12'N	11°27'E	2180	2007	PICE	L	5	1.5	26
DRY	AT-Tschirgant dry-mesic	T	47°14'N	10°50'E	750	2010-2011	LADE, PCAB, PISY	E, L, E	29	9.0	27
SIM2	CA-Simoncouche2	B	48°12'N	71°14'W	350	2010-2011	ABBA, PCMA	L	12	2.6	28
SIM	CA-Simoncouche	B	48°13'N	71°15'W	338	2005-2014	ABBA, PCMA	L	55	2.4	29
ABR	FR-Abreschviller	T	48°21'N	7°4'E	430	2007-2009	ABAL, PCAB, PISY	L, L, E	16	9.2	31
WAL	FR-Walscheid	T	48°22'N	7°5'E	370	2007-2009	ABAL, PCAB, PISY	L, L, E	16	10.3	31
ARV	CA-Arvida	B	48°26'N	71°9'W	80	1999-2000	ABBA	L	18	3.7	19
GRA	FR- Grandfontaine	T	48°28'N	4°08'E	650	2007-2008	ABAL, PCAB, PISY	L, L, E	15	8.2	31
GRD	FR-Grandfontaine	T	48°29'N	7°9'E	643	2007-2009	ABAL, PCAB, PISY	L, L, E	15	8.6	31
AMA	FR-Amance forest	T	48°44'52"N	6°19'30"E	270	2006-2007	ABAL	L	20	10.5	32
AMA*	FR-Amance forest	T	48°44'52"N	6°19'30"E	270	2006-2007	PISY	E	25	10.4	33
BER	CA-Bernatchez	B	48°51'N	70°20'W	611	2002-2014	PCMA	L	31	0.5	29
SOB*	CZ-Brno	T	49°09'N	16°22'W	404	2013-2014	PISY	E	6	10.5	34

ID	Site	Biome	Latitude	Longitude	Altitude (m a.s.l.)	Study years	Species	Successional stage	Number of trees	Site temperature(°C)	References
RAJ	CZ-Drahany highland	T	49°26'N	16°41'W	620	2009-2011	PCAB	L	6	8.1	21
MIS	CA-Mistassibi	B	49°43'N	71°56'W	342	2002-2014	PCMA	L	28	1.1	29
O1	DE-Bayreuth	T	49°55'N	11°35'W	355	2013	CELI	L	3	8.2	7
L23	CA-Liberal 23	B	49°58'N	72°30'W	380	1998-2000	ABBA	L	10	1.5	35
L24	CA-Liberal 24	B	49°58'N	72°30'W	430	1998-2001	ABBA	L	20	1.0	35
DAN	CA-Camp Daniel	B	50°41'N	72°11'W	487	2002-2014	PCMA	L	25	-0.7	29
LH1	CZ-Lucní Hora-timberline	T	50°43'N	15°40'E	1310	2010-2012	PCAB	L	10	3.4	36
LH2	CZ-Lucní Hora-treeline	T	50°43'N	15°41'E	1450	2010-2012	PCAB	L	10	1.8	36
BLS	CZ-Bílé Labe Valley north	T	50°44'N	15°39'E	1270	2013-2014	PCAB	L	8	3.9	11,22
BLJ	CZ-Bílé Labe Valley south	T	50°44'N	15°39'E	1270	2013-2014	PCAB	L	8	4.2	11, 22
SISE	CZ-Maly Sisak east	T	50°45'N	15°39'E	1375	2014-2015	PCAB	L	8	4.2	37
SISW	CZ-Maly Sisak west	T	50°46'N	15°38'E	1360	2014-2015	PCAB	L	8	4.1	37
MIR	CA-Mirage	B	53°47'N	72°52'W	384	2012-2014	PCMA	L	10	-2.3	38
RUO*	FI-Ruotsinkylä	B	60°12'N	25°0'E	60	2008-2010	PCAB, PISY	L, E	15	5.6	39
HYY*	FI-Hyytiälä	B	61°53'N	24°18'E	181	2008	PCAB, PISY	L, E	6	4.9	40
KIV*	FI-Kivalo	B	66°12'N	26°23'E	140	2009	PCAB	L	5	1.1	40
VAR*	FI-Värriö	B	67°30'N	29°23'E	390	2009	PISY	E	4	0.2	40

Table S2. Statistics of Models: DOY ~ Photoperiod, and DOY ~ MAT, where photoperiod and MAT were fixed effects in the two models. Values in brackets represent the total number of records. Note: For fixed effects, standard error (SE) is given in brackets; P<0.001 (***); P<0.01(**); P<0.05 (*); Marginal R^2 (fixed effects only), Conditional R^2 (both fixed and random effects); AIC (Akaike information criterion); BIC (Bayesian information criterion); DOY: day of the year; MAT: mean annual temperature.

	Photoperiod	MAT	Variance explained
Fixed effects			
Intercept	-218.91(5.65)***	160.10(3.49)***	
Photoperiod	23.85(0.31)***		46%
MAT		-4.09(0.23)***	50%
Random effects			
SD(site)	30.47	9.68	
SD(species)	1.57	12.24	
SD(residual)	5.25	10.06	
Model fit			
Marginal R^2	0.46	0.50	
Conditional R^2	0.98	0.85	
AIC	13001	15408	
BIC	13029	15436	

Table S3: Statistics of Model: DOY ~ MAT + Forcing + Chilling + Prep. Note: For fixed effects, standard error (SE) is given in brackets; P<0.001 (***); P<0.01(**); P<0.05 (*); Marginal R^2 (fixed effects only), Conditional R^2 (both fixed and random effects); AIC (Akaike information criterion); BIC (Bayesian information criterion); DOY: day of the year, the onset date of wood formation; MAT: mean annual temperature; Prep: precipitation; Number of observation =1480, which is lower than other models without the variable of precipitation due to missing values in precipitation data. Variance explained for the model with precipitation from January 1st to DOY was listed below.

	**Temperature + Forcing + Chilling+ Prep**		
	Annual total precipitation	Precipitation (January 1 to DOY)	Variance explained
**Fixed effects**			
Intercept	121.4(2.35)***	115.7(2.09)***	
Temperature	-5.97(0.16)***	-5.65(0.16)***	46.6%
Forcing	0.11(0.002)***	0.11(0.002)***	31.2%
Chilling	0.29(0.01)***	0.27(0.01)***	4.5%
Prep.	-0.0008(0.001)	0.02(0.002)*	0.0%
**Random effects**			17.6%
SD(site)	9.31	9.81	
SD(species)	2.33	1.56	
SD(residual)	5.50	5.39	
Model fit			
Marginal R^2	0.84	0.82	
Conditional R^2	0.96	0.96	
AIC	9544	9480	
BIC	9586	9523	

Table S4: Comparison among different models based on different SPEI temporal scales. Statistics of Model: DOY ~ MAT + Forcing + Chilling + SPEI. Note: For fixed effects, standard error (SE) is given in brackets; P<0.001 (***); P<0.01(**); P<0.05 (*); Marginal R^2 (fixed effects only), Conditional R^2 (both fixed and random effects); AIC (Akaike information criterion); BIC (Bayesian information criterion); DOY: day of the year; MAT: mean annual temperature. SPEI: Standard Precipitation-Evapotranspiration Index.

	**Temperature + Forcing + Chilling+SPEI**		
	1-month SPEI	3-month SPEI	6-month SPEI
**Fixed effects**			
Intercept	127.0(1.70)***	125.7(1.63)***	125.9(1.67)***
Temperature	-6.08(0.13)***	-5.87(0.13)***	-5.97(0.13)***
Forcing	0.12(0.002)***	0.11(0.002)***	0.12(0.002)***
Chilling	0.22(0.01)***	0.22(0.01)***	0.22(0.01)***
SPEI	1.47(0.12)***	2.17(0.13)***	2.44(0.16)***
**Random effects**			
SD(site)	7.47	7.69	7.59
SD(species)	2.42	1.91	2.31
SD(residual)	5.85	5.68	5.72
Model fit			
Marginal R^2	0.84	0.83	0.84
Conditional R^2	0.94	0.94	0.94
AIC	13151	13041	13067
BIC	13195	13086	13112

Table S5: Model results of Mediterranean biome. Statistics of Model 2: DOY ~ MAT + Forcing + Chilling + scPDSI. Note: For fixed effects, standard error (SE) is given in brackets; P<0.001 (***); P<0.01(**); P<0.05 (*); Marginal R^2 (fixed effects only), Conditional R^2 (both fixed and random effects); AIC (Akaike information criterion); BIC (Bayesian information criterion); DOY: day of the year; MAT: mean annual temperature. scPDSI: the self-calibrating Palmer Drought Severity Index.

	**Model 2: MAT + Forcing + Chilling+scPDSI**	P value	Variance explained
Fixed effects			
Intercept	122.3 (7.32)***	<0.0001	
MAT	-4.8 (0.34)***	<2e-16	17.08%
Forcing	0.12(0.01)***	<2e-16	10.46%
Chilling	0.11(0.05)	0.059	13.77%
scPDSI	-0.56(0.30)	0.059	9.61%
Random effects			44.59%
SD(site)	12.4		
SD(species)	11.9		
SD(residual)	5.5		
Model fit			
Marginal R^2	0.51		
Conditional R^2	0.96		
AIC	1284		
BIC	1310		

Table S6: Statistics of Model 2 at different chilling thresholds. MAT: mean annual temperature.

Variables	MAT	Forcing	Chilling (0 ℃ to 5 ℃)	scPDSI	Random	Unexplained
Variance explained (%)	52.3	22.09	5.63	0.00	13.12	6.87
Variables	MAT	Forcing	**Chilling (-5 ℃ to 5 ℃)**		Random	Unexplained
Variance explained (%)	52.85	22.92	**8.47**	0.17	10.07	5.51
Variables	MAT	Forcing	Chilling (-5 ℃ to 0 ℃)		Random	Unexplained
Variance explained (%)	52.09	22.68	6.11	0.0	12.90	6.23
Variables	MAT	Forcing	Chilling (-10 ℃ to 0 ℃)		Random	Unexplained
Variance explained (%)	52.04	22.13	5.33	0.00	14.95	5.54

Dataset S1: Data that support the findings of this study are available in Data.csv.

References

1. H. Hänninen, Modeling bud dormancy release in trees from cool and temperate regions. *Acta Forestalia Fennica* 213. The Society of Forestry in Finland, The Finnish Forest Research Institute, Helsinki (1990).
2. H. Hänninen *et al.*, Experiments are necessary in process-based tree phenology modelling. *Trends Plant Sci.* **24(3)**, 199–209 (2019).
3. Y.H. Fu *et al.*, Declining global warming effects on the phenology of spring leaf unfolding. *Nature* **526**, 104–107 (2015).
4. J.G. Huang, *et al.* Intra-annual wood formation of subtropical Chinese red pine shows better growth in dry season than wet season. *Tree Physiol.* **38**, 1225–1236 (2018).
5. X. Li, E. Liang, J. Gricar, P. Prislan, S. Rossi, K. Cufar, Age-dependence of xylogenesis and its climatic sensitivity in Smith fir on the south-eastern Tibetan Plateau. *Tree Physiol.* **33**, 48–56 (2013).
6. S.K. Zhang *et al.*, Intra-annual dynamics of xylem growth in *Pinus massoniana* under an experimental N addition in central China. *Tree Physiol.* **37**, 1546–1553 (2017).
7. A. Güney, D. Kerr, A. Sökücü, R. Zimmermann, M. Küppers, Cambial activity and xylogenesis in stems of *Cedrus libani* A. Rich at different altitudes. *Bot. stud.* **56(1)**, 20 (2015).
8. E. Ziaco, C. Truettner, F. Biondi, S. Bullock, Moisture-driven xylogenesis in *Pinus ponderosa* from a Mojave Desert mountain reveals high phenological plasticity. *Plant, Cell Environ.* **41 (4)**, 823–836 (2018).
9. Q. Zeng, S. Rossi, B. Yang, Effects of age and size on xylem phenology in two conifers of Northwestern China. *Front. Plant. Sci.* **8**, 2264 (2017).
10. S. Rossi, *et al.*, Pattern of xylem formation in conifers of cold ecosystems at the Northern Hemisphere. *Global Change Biol.* **22**, 3804–3813 (2016).
11. H.E. Cuny *et al*., Wood biomass production lags stem-girth increase by over one month in coniferous forests. *Nature Plants* **15160**, (2015).
12. S. Antonucci, S. Rossi, F. Lombardi, M. Marchetti, R. Tognetti, Influence of climatic factors on silver fir xylogenesis along the Italian peninsula. *IAWA* **40**, 259–275 (2019).
13. E. Ziaco, F. Biondi, Tree growth, cambial phenology, and wood anatomy of limber pine at a Great Basin (USA) mountain observatory. *Trees* **30(5)**, 1507-1521 (2016).
14. E. Ziaco, F. Biondi, S. Rossi, A. Deslauriers, Environmental drivers of cambial phenology in Great Basin bristlecone pine. *Tree Physiol.* **36(7)**, 818-831 (2016).
15. A. Deslauriers, S. Rossi, T. Anfodillo, A. Saracino, Cambium phenology, wood formation and temperature thresholds in two contrasting years at high altitude in Southern Italy. *Tree Physiol.* **28**, 863–871 (2008).
16. J. Vieira *et al*., Adjustment capacity of maritime pine cambial activity in drought-prone environments. *PLOS ONE* **10(5)**, e0126223 (2015).

17. J.J. Camarero, G. Guada, R. Sánchez-Salguero, E. Cervantes, Winter drought impairs xylem phenology, anatomy and growth in Mediterranean Scots pine forests. *Tree Physiol*. **36**, 1536–1549 (2016).
18. J.J. Camarero, J.M. Olano, A. Parras, Plastic bimodal xylogenesis in conifers from continental Mediterranean climates. *New Phytol.* **185**, 471–480 (2010).
19. S. Rossi *et al*., Conifers in cold environments synchronize maximum growth rate of tree-ring formation with day length. *New Phytol.* **170**, 301–310 (2006).
20. C. Cocozza *et al*., Monitoring intra-annual dynamics of wood formation with microcores and dendrometers in *Picea abies* at two different altitudes. *Tree Physiol.* **36**, 832–846 (2016).
21. J. Gričar, P. Prislan, V. Gryc, H. Vavrčík, M. de Luis, Plastic and locally adpated phenology in cambial seasonality and production of xylem and phloem cells in *Picea abies* from temperate environments. *Tree Physiol.* **34**, 869–881 (2014).
22. H.E. Cuny *et al*., Couplings in cell differentiation kinetics mitigate air temperature influence on conifer wood anatomy. *Plant, Cell Environ.* **42**, 1222–1232 (2019).
23. T. Anfodillo *et al.*, Widening of xylem conduits in a conifer tree depends on the longer time of cell expansion downwards along the stem. *J. Exp. Bot.* **63**, 837–845 (2012).
24. S. Rossi, A. Deslauriers, T. Anfodillo, V. Carraro, Evidence of threshold temperatures for xylogenesis in conifers at high altitude. *Oecologia* **152**, 1–12 (2007).
25. S. Rossi, A. Deslauriers, T. Anfodillo, M. Carrer, Age-dependent xylogenesis in timberline conifers. *New Phytol.* **177**, 199–208 (2008).
26. A. Gruber, G. Wieser, W. Oberhuber, Intra-annual dynamics of stem CO_2 efflux in relation to cambial activity and xylem development in *Pinus cembra*. *Tree Physiol.* **29**, 641–649 (2009).
27. I. Swidrak, A. Gruber, W. Oberhuber, Xylem and phloem phenology in co-occurring conifers exposed to drought. *Trees* **28**, 1161–1171 (2014).
28. A. Lemay, C. Krause, S. Rossi, A. Achim, Xylogenesis in stems and roots after thinning in the boreal forest of Quebec, Canada. *Tree physiol.* **37**, 1554–1563 (2017).
29. S. Rossi, H. Morin, A. Deslauriers, P.Y. Plourde, Predicting xylem phenology in black spruce under climate warming. *Global Change Biol.* **17**, 614–625 (2011).
30. H.E. Cuny, C.B. Rathgeber, D. Frank, P. Fonti, M. Fournier, Kinetics of tracheid development explain conifer tree-ring structure. *New Phytol.* **203**, 1231–41 (2014).
31. C.B.K. Rathgeber, S. Rossi, J.D. Bontemps, Cambial activity related to tree size in a mature silver-fir plantation. *Ann. Bot.* **108**, 429–438 (2011).
32. C.B.K. Rathgeber, F. Longuetaud, F. Mothe, H. Cuny, G.L. Moguédec, Phenology of wood formation: Data processing, analysis and visualisation using R (package CAVIAR). *Dendrochronologia* **29**, 139–149 (2011).
33. M. Fajstavr, K. Giagli, H. Vavrčík, V. Gryc, J. Urban, The effect of stem girdling

on xylem and phloem formation in Scots pine. *Silva Fennica* vol. **51** no. 4 article id 1760. 22p. (2017).

34. A. Deslauriers, H. Morin, Y. Begin, Cellular phenology of annual ring formation of *Abies balsamea* in the Quebec boreal forest (Canada). *Can. J. For. Res*. **33(2)**, 190–200 (2003).
35. V. Treml, J. Kašpar, H. Kuželová, V. Gryc, Differences in intra-annual wood formation in *Picea abies* across the treeline ecotone, Giant Mountains, Czech Republic. *Trees* **29**, 515–526 (2015).
36. V. Treml, T. Hejda, J. Kašpar, Differences in growth between shrubs and trees: How does the stature of woody plants influence their ability to thrive in cold regions? *Agric. For. Meteorol.* **271**, 54–63 (2019).
37. S. Rossi, M.J. Girard, H. Morin, Lengthening of the duration of xylogenesis engenders disproportionate increases in xylem production. *Global Change Biol.* **20**, 2261–2271 (2014).
38. H. Mäkinen, T. Jyske, P. Nöjd, Dynamics of diameter and height increment of Norway spruce and Scots pine in southern Finland. *Ann. For. Sci.* **75(1)**, 28. (2018).
39. T. Jyske, H. Mäkinen, T. Kalliokoski, P. Nöjd, Intra-annual xylem formation of Norway spruce and Scots pine across latitudinal gradient in Finland. *Agric. For. Meteorol*. **194**, 241–254 (2014).
